# Associations between Attention and Implicit Associative Learning in Healthy Adults: The Role of Cortisol and Salivary Alpha-Amylase Responses to an Acute Stressor

**DOI:** 10.3390/brainsci10080544

**Published:** 2020-08-12

**Authors:** Linda Becker, Nicolas Rohleder

**Affiliations:** Department of Psychology, Friedrich-Alexander University Erlangen-Nürnberg, 91052 Erlangen, Germany; nicolas.rohleder@fau.de

**Keywords:** stress, cognition, cortisol, alpha-amylase, implicit learning, associative memory, attention, Stroop, SECPT

## Abstract

In this study, we investigated the associations between implicit associative learning with the cortisol and salivary alpha-amylase (sAA) stress response to an acute stressor as well as their associations with attention. Eighty one healthy adults (25 male) participated and either performed the socially evaluated cold-pressor test (SECPT) or a warm-water control task (WWT). Either prior to or immediately after the SECPT/WWT, participants implicitly learned digit-symbol pairs. A not-previously announced recall test was conducted about 20 min after the SECPT/WWT. Attention was assessed by means of a Stroop task at nine time points over the course of the experiment. Memory recall performance was not significantly associated with the acquisition time point (pre or post stressor) and did not significantly differ between the responder groups (i.e., non-responders, sAA-and-cortisol responders, only sAA responders, and only cortisol responders). Attentional performance increased throughout the experiment (i.e., reaction times in the Stroop task decreased). No differences in the attentional time course were found between the responder groups. However, some associations were found (*p*_uncorrected_ < 0.05) that did not pass the multiple comparison adjusted alpha level of α_adjusted_ = 0.002, indicating different associations between attention and implicit learning between the responder groups. We conclude that the associations of sAA and cortisol responses with implicit learning are complex and are related to each other. Further studies in which both (sAA and cortisol responses) are selectively (de-) activated are needed. Furthermore, different learning tasks and less—potentially stressful—attentional assessments should be used in future research. Moreover, field studies are needed in which the associations between acute stress and implicit associative learning are investigated in everyday life.

## 1. Introduction

One of the most important cognitive functions for a successful and happy life is memory. Memory is much more than remembering the names of loved ones or telephone numbers. It can also be the result of learning new skills or associations, which mostly happens unconsciously. This unconsciously learned information is called implicit memory, the opposite of explicit memory [1]. Another distinction that has often been made, is whether the hippocampus is involved in the learning process or not, which leads to so-called hippocampus-dependent or non-hippocampus-dependent memory [2]. Hippocampus-dependent memory includes both explicit memory as well as some forms of implicit memory (e.g., associative memory; [3,4,5]).

Implicit learning and memory can be affected by stress (e.g., [6,7,8]). This can have far-reaching consequences if, in the long term, new skills are not being learned or are being learned incorrectly. The human stress response is associated with the activation of different physiological pathways (e.g., [9,10]). The first, fast stress response is the activation of the sympathetic nervous system (SNS), which leads to the release of the catecholamines epinephrine and norepinephrine. The second, slower response is the activation of the hypothalamic-pituitary adrenal (HPA) axis, which leads to the release of glucocorticoids (i.e., cortisol in humans). Both catecholamines and glucocorticoids can affect cognitive processing through direct and indirect pathways [11,12,13]. With regard to learning and memory, the HPA axis response is of particular importance, because glucocorticoids can bind to receptors in brain structures which are associated with memory formation (e.g., the hippocampus; [14,15]). Stress and the related release of catecholamines and glucocorticoids was found to have both positive and negative effects on human memory, depending on several factors such as the stimulus material (e.g., neutral vs. emotional; [16,17]) or the type of memory (e.g., implicit vs. explicit, hippocampus-dependent vs. non-hippocampus-dependent; [18,19,20]). An additional crucial factor is the time point of the stressor (i.e., during acquisition/learning, consolidation, or retrieval). In most previous studies, it has been found that stress during the acquisition phase can improve learning and memory [21,22,23]. However, in these studies, explicit memory tasks have mostly been used. With regard to the associations between stress and memory retrieval, previous findings point in the opposite direction, i.e., it has been found that particularly explicit memory retrieval is impaired by acute stress (e.g., [24,25,26,27,28]).

Overall, most studies that examine the association between stress and memory have used explicit rather than implicit memory tasks and less is known about the associations between stress and implicit memory. The findings with respect to implicit learning and memory are divergent: Meyer and colleagues (2013) found an improvement in a spatial contextual cueing task for high-cortisol responders and a performance decrease in low-cortisol responders [8]. On the other hand, Dinse and colleagues (2017) found that elevated glucocorticoid levels were associated with a blockade of human tactile perceptual learning [29]. Luethi and colleagues (2009) found that stress enhanced classical conditioning for negative stimuli which was not found for positive ones [6]. Therefore, the direction of the associations between implicit learning and the stress response is still under debate and the underlying physiological mechanisms are not yet fully understood.

All the above-mentioned factors, which are related to the association between stress and memory (i.e., stimulus material, type of memory, and time point of the stressor), are only a small selection of factors and many more could be possible. One, which has been neglected in most cases so far, is the participant’s general cognitive functioning, including, for example, attention, working memory, and processing speed. These cognitive functions can be associated with the stress response too, which further complicates the situation (e.g., [30,31,32,33,34]). A crucial cognitive factor for the formation of memories is attention because it supports the filtering of relevant and the rejection of irrelevant information [35]. Although often happening unconsciously, attention is also necessary for implicit learning and memory [36,37,38,39]. The associations between stress and attention are also complex and are differentially related with the SNS and HPA axis stress response. In several previous studies, an improvement in selective attention for relevant information and an impairment for irrelevant information, related to the catecholamine response, have been reported [40,41,42]. The association between attention and the glucocorticoid response rather points in the opposite direction, i.e., higher glucocorticoid levels were associated with lower attentional performance [21,43,44,45].

In our study, we investigated whether implicit associative learning and memory is associated with SNS or HPA axis stress responses, i.e., with the release of either catecholamines or glucocorticoid or both. We hypothesized that implicit memory recall would be lower in participants who show a glucocorticoid (i.e., cortisol) response than in cortisol-non-responders. Furthermore, we compared implicit memory performance between participants for whom the acquisition phase preceded the stressor/control and participants who performed the memory acquisition after the stressor/control task. In accordance with previous studies [21,22,23], we hypothesized that acute stress immediately before acquisition would improve consolidation and that, therefore, implicit memory recall performance would be better in the post-stress than in the pre-stress group. Furthermore, we investigated the time course of attentional performance in response to the stressor and its associations with the stress response and with implicit learning.

Our study should enable us to better understand the underlying processes and the timing of the association between stress and implicit learning. In addition, a better understanding of whether attention can be a potential protective factor should be enabled by our study.

## 2. Materials and Methods

### 2.1. Participants

Participants were *N* = 81 healthy adults (25 male, mean age = 21.8 ± 3.9 years, BMI = 22.1 ± 2.8 kg/m^2^, and two smokers of fewer than five cigarettes a day). An overview of the participants’ demographics is provided in Table 1. Seventy-six were students from the Friedrich-Alexander University Erlangen-Nürnberg (FAU). The other participants were employees from the same region. Exclusion criteria were usage of beta-blockers or glucocorticoid medication, physical or psychological diseases, smoking more than five cigarettes a day, color blindness, or dyschromatopsia. From initially *N* = 91 participants, two were excluded because they did not understand the Stroop task correctly and showed error rates greater than 50%, one was excluded because of technical problems, and seven were excluded because they did not provide enough saliva for analysis. All participants gave their written and informed consent. Data was anonymized directly after collection to protect participants’ privacy. The study was conducted according to the principles expressed in the Declaration of Helsinki and was approved by the local ethics committee of the FAU (#6_18 B).

### 2.2. Cognitive Testing

**Figure 1:**
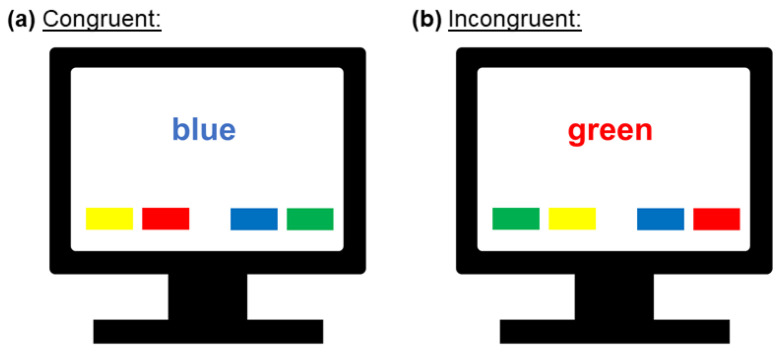
Conditions in the Stroop task: (**a**) congruent; (**b**) incongruent. Each block consisted of 40 (13 congruent, 27 incongruent) trials that were displayed in a randomized order.

**Figure 2:**
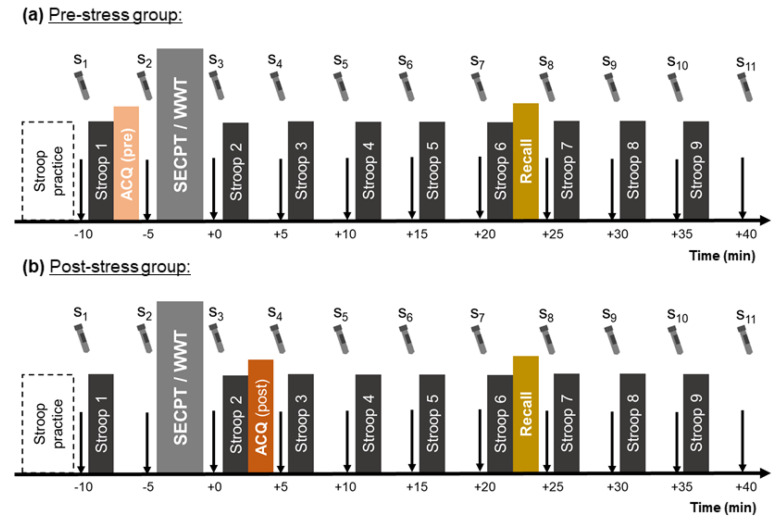
Experimental procedure. The memory acquisition (ACQ) phase was either performed (**a**) before or (**b**) after the socially evaluated cold-pressor (SECPT) or the warm-water test (WWT).

Attention was assessed by means of a computerized version of the color-word Stroop test [46]. The stimuli consisted of the German words for yellow, blue, red, or green that were either displayed in a congruent or an incongruent color. Under the words, colored squares in the four colors were presented and assigned to the response buttons (letters Y, X, N, and M on the keyboard; Figure 1). Participants were instructed to press the button that corresponds to the print color of the word and to ignore the word’s semantics. Each block consisted of 40 (13 congruent and 27 incongruent) trials that were displayed in a randomized order. The ratio of 33% incongruent trials was chosen because we did not want to make the task too easy (e.g., for 50% congruent trials) nor did we want to have too little a number of congruent trials. Nine blocks of the Stroop task were performed by each participant, one before the SECPT/WWT and eight after it (Figure 2). Stimuli were presented until a response was made, but no longer than 2500 ms. Inter-stimulus intervals were set to 500 ms. For evaluation, the first three trials of each block were discarded and mean reaction times (RT) for the remaining 37 trials and number of errors were computed. Reaction times and the number of errors were used as attentional performance measures, i.e., longer reaction times and higher error rates were associated with poorer attentional performance. The number of errors is labeled as “# Errors Stroop“ in the following. 

In the learning phase (before or after the stressor/control), a part of the digit-symbol-substitution test (DSST), which is part of the Wechsler adult intelligence scale (WAIS; [47]), was filled out by the participants. The task is to assign a symbol to the digits 1 to 9 according to a legend. The processing time was 60 s. The DSST was introduced as an additional cognitive test to the participants with the instruction to work as fast and as accurately as possible. The participants were not made aware that this task was part of an implicit memory test. The number of correctly processed items was used as a performance measure during the acquisition phase. Because only one error was made by a single participant, the number of errors was not evaluated. Without being announced beforehand, participants were asked to recall the digit-symbol pairs 20 min after the stressor/control. Participants were given up to 60 s for the recall. The numbers of errors (labeled as “# Errors recall“) and the numbers of correctly recalled items (labeled as “# Correct recall“) were used as recall performance measures. The maximum number of correctly learned associations was nine.

### 2.3. Stress Induction and Control Condition

For stress induction, a group version of the SECPT [48,49,50] was used. Participants met in pairs of two in the experimental room and were placed around a large table with transparent boxes filled with ice water in front of them. They were instructed to immerse their hands in the water, which had a temperature between 2 and 3 °C as long as possible for up to three minutes. Mean immersion time was 2:30 ± 0:42 min (maximum: 3:00 min, minimum: 0:33 min). The hand of each participant was directly opposite to the hand of the other person with the aim to introduce a competitive situation. Remaining time was displayed on a large-display digital clock that was visible to both participants. An auditory countdown announced the last five seconds. Furthermore, participants were videotaped during the test. One experimenter was present during the SECPT who was instructed to behave distanced and to have a neutral mimic. As a control condition, a warm-water test (WWT) was performed in which the participants were instructed to immerse their hand in warm water (30 ± 2 °C). The set-up was similar to the SECPT, but the participants were not videotaped. Based on our experience with response rates from previous studies [32,48] and with the goal to achieve approximately equal group sizes, *N* = 69 participants conducted the SECPT and *N* = 12, the WWT.

### 2.4. Saliva Sampling and Analysis

Catecholamine transmission was indirectly assessed by measuring salivary alpha-amylase (sAA) levels. Salivary-alpha amylase is a well-suited marker, because sAA levels are highly correlated with blood-norepinephrine levels in humans [51,52] and are, therefore, a suitable, non-invasive marker. Glucocorticoid transmission was assessed by means of salivary cortisol samples. Saliva samples were collected by means of salivettes (Sarstedt, Nümbrecht, Germany). Participants were instructed to keep the salivette in their mouth for at least one minute and to move it back and forth, but not to chew on it. During each saliva sampling, the current level of perceived stress was rated on 10-point Likert scales with the anchors “not stressed at all” and “extremely stressed”. Saliva samples were stored at −30 °C immediately after collection. For analysis, salivettes were thawed at room temperature and were centrifuged at 2000 *g* and 20 °C for ten minutes immediately before analysis. Salivary α-amylase was measured with an in-house enzyme kinetic assay using reagents from DiaSys Diagnostic Systems GmbH (Holzheim, Germany), as previously described [53,54]. In brief, saliva was diluted at 1:625 with ultrapure water, and diluted saliva was incubated with substrate reagent (α-amylase CC FS; DiaSys Diagnostic Systems) at 37 °C for three minutes before a first absorbance reading was taken at 405 nm with a Tecan Infinite 200 PRO reader (Tecan, Crailsheim, Germany). A second reading was taken after five minutes incubation at 37 °C and increase in absorbance was transformed to sAA concentrations (U/mL), using a standard curve prepared using “Calibrator f.a.s.” solution (Roche Diagnostics). Salivary cortisol concentrations were determined in duplicate using chemiluminescence immunoassay (CLIA, IBL, Hamburg, Germany). Intra- and inter-assay coefficients of variation were below 10% for both sAA and cortisol.

### 2.5. General Procedure

The study was conducted between October 2018 and June 2019. The experimental sessions started between 1 and 8 p.m. The participants were not allowed to eat, smoke, or drink (except water) for at least one hour prior to and during the experiment. After being informed about the experimental procedure, participants gave their written consent for participation. The participants were informed that they would perform several cognitive tests throughout the session and that they might become stressed for a few minutes. Participants were not made aware of the condition to which they were assigned. Furthermore, participants were not made aware that a memory task would occur. At the beginning of the session, two practice trials of the Stroop task were performed. After this, the first saliva sample (s_1_) was collected and the first experimental trial of the Stroop task was performed. Then, *N* = 37 of the participants (the pre-stress group) performed the DSST (i.e., the implicit memory acquisition phase). After this, participants were brought to another room where the next saliva sample (s_2_) was taken (5 min after s_1_). Then, the SECPT or WWT was instructed to the participants and was then started. Immediately after the SECPT/WWT, the third saliva sample (s_3_) was collected. After this, participants went back to the previous room and either performed the next Stroop trial, followed by the DSST (the post-stress group, *N* = 44) or the Stroop trial only. Subsequently, the next saliva samples were collected every five minutes and the further Stroop trials were conducted in-between. The recall phase of the memory test was timed about 20 min after the stressor/control where the cortisol levels were expected to be highest in the only-cortisol- and the sAA-and-cortisol-responders. The DSST recall had not been announced before, i.e., participants were not made aware that they would be doing a memory test during the experiment. The experiment ended after the eleventh saliva sample was collected (40 min after the stressor or control task). The whole session lasted about 60 min (Figure 2).

### 2.6. Statistical Data Analysis

For statistical analyses, IBM SPSS Statistics (version 26) was used. Normality of distribution was tested by means of the Kolmogorov–Smirnov test. Because of positive skewness and violation of normality, sAA and cortisol levels were transformed by means of the natural logarithm (ln) prior to further statistical analysis. Participants were categorized as (only-) sAA-responders if they showed an sAA increase of more than 10% and of at least 10 U/mL between s_2_ (before SECPT/WWT) and s_3_ (immediately after SECPT/WWT). A cortisol increase of more than 10% and of at least 1 nmol/L between s_2_ and s_7_ (20 min after the SECPT/WWT) was used as a criterion for being categorized as (only-) cortisol-responders. These criteria have been proven to be suitable to distinguish responders from non-responders in previous studies in our group [32] and are slightly lower than criteria that were reported by other authors (e.g., 1.5 nmol/L and 15.5% for cortisol; [55,56]). Participants who fulfilled both criteria were categorized as sAA-and-cortisol-responders and participants who fulfilled none were classified as non-responders. These classifications were independent of whether the SECPT or WWT was performed. The area under the curve with respect to ground (AUCg; [57]) was calculated between s_2_ and s_4_ for sAA and between s_2_ and s_7_ for cortisol as measures for total sAA and cortisol outputs. We did not use the area under the curve with respect to increase (AUCi; [57]), because the differentiation between decreases and increases was already taken into account by the different responder groups.

Analyses of variance for repeated measurements (rmANOVAs) with the within-subject factor “time” (s_1_–s_11_) and the between-subjects factor “task” (WWT vs. SECPT) were calculated separately for perceived stress ratings, sAA, and cortisol. For post-hoc analyses, comparisons between s_1_ (at baseline) and s_3_ (immediately after the stressor/control) for perceived stress, between s_2_ (immediately before the WWT/SECPT) and s_3_ for sAA and between s_1_ and s_7_ (20 min after the stressor) for cortisol were performed. These time points were chosen because we wanted to specifically test whether the difference between the baseline level (which was lowest at s_1_ for perceived stress and at s_2_ for sAA and cortisol) and the time point of the expected peak after the stressor/control (i.e., at s_3_ for perceived stress and sAA and 20 min after the stressor for cortisol) was significant. We did not conduct all possible post-hoc comparisons because they were not relevant for the research question. For evaluation of the Stroop performance, additional rmANOVAs with the within-subject factor “time” (Stroop 1–Stroop 9) and the between-subjects factor “responders” were calculated. Partial eta-squares (η_p_^2^) were considered as effect sizes. Sphericity was tested by means of the Mauchly test [58]. If necessary, degrees of freedom were corrected by means of the Greenhouse–Geisser procedure [59]. For post-hoc analyses, *t*-tests for dependent samples were calculated and Cohen’s *d* was considered as a measure for effect sizes. Cohen’s *d* was corrected according to the method that was proposed by Morris [60]. For comparisons of learning and recall performance between the pre- and the post-stress-group, *t*-tests for independent samples were calculated. Recall performance between the responder groups was compared by means of univariate analyses of variance (ANOVA). Alpha-levels of 0.05 were used for these statistical analyses. For *t*-tests, 95% confidence intervals (CI) are reported. To investigate whether the stress response or Stroop performance was associated with acquisition or recall performance, bivariate Pearson correlations *r* were calculated. For these analyses, the percentage and total sAA and cortisol increase and AUCgs were used as markers for the stress response. We decided to use both the total and the percentage increase, because both provide different information. The percentage change primarily gives information on how strong a change is within a person in dependence of the individual baseline level. The absolute increase allows a better assessment of the importance of the increase. The AUCg enables the total amount of the released sAA and cortisol to be estimated and, thus, provides additional information. The correlation analyses were conducted for the whole sample as well as separately for the pre- and post-stress group, and for the four responder groups. To correct for multiple comparisons, a Bonferroni-adjusted α-level of α_adjusted_ = 0.05/(6 × 4) = 0.05/24 = 0.002 was used for the correlation analyses, because six physiological markers and four responder groups were compared [61]. However, because making decisions based on (non-) significance only has been increasingly criticized and it has been becoming more and more common to interpret effect sizes instead of *p*-values (e.g., [62]), we still report results which fulfill the criterion *p*_uncorrected_ ≤ 0.05. We consider correlation coefficients *r* between 0.1 and 0.2 as small effects, between 0.2 and 0.3 as medium effects, and >0.3 as large effects [63].

## 3. Results

### 3.1. Baseline Characteristics

An overview of participants’ baseline demographic characteristics and baseline levels of subjective stress ratings, sAA, and cortisol levels as well as baseline levels of attentional performance, are provided in Table 1. None of the baseline variables (i.e., demographics, subjective ratings, physiological variables, and cognitive performance) differed between the SECPT and the WWT group (all *p* ≥ 0.097). Furthermore, no significant differences in any of the baseline characteristics between participants from the pre- and the post-stress group were found (all *p* ≥ 0.304).

### 3.2. Stress Response

**Figure 3:**
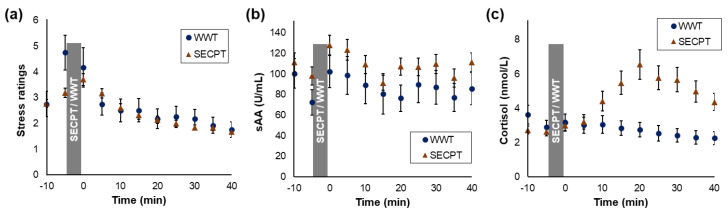
Time course of perceived stress ratings (**a**), sAA (**b**), and cortisol levels (**c**), separately for the socially evaluated cold-pressor test (SECPT; *N* = 69) and the warm-water test (WWT; *N* = 12) groups. Standard errors are shown as error bars.

For perceived stress, a main effect of the factor time (*F*_(4.2, 327.8)_ = 26.629, *p* < 0.001, η_p_^2^ = 0.25) and an interaction time × task (*F*_(4.5, 79)_ = 2.82, *p* = 0.020, η_p_^2^ = 0.03) were found. Separate rmANOVAs for both groups indicated that perceived stress significantly changed during the experiment in both the SECPT (*F*_(4.7, 317.2)_ = 34.18, *p* < 0.001, η_p_^2^ = 0.34) and in the WWT groups (*F*_(2.8, 30.6)_ = 9.75, *p* < 0.001, η_p_^2^ = 0.47; Figure 3a). However, post-hoc analyses indicated that perceived stress was significantly higher after the task than before in the SECPT group only (s_1_–s_3_: *t*_(69)_ = −4.11, *p* < 0.001, *d* = 0.65, CI (−1.38, −0.48)), but did not significantly change in the WWT group (s_1_–s_3_: *p* = 0.101, *d* = 0.69).

For sAA, a main effect of time (*F*_(6.6, 521.3)_ = 4.68, *p* < 0.001, η_p_^2^ = 0.06), but no interaction time × task (*p* = 0.901, η_p_^2^ = 0.01) was found, indicating that sAA levels significantly changed in both groups (Figure 3b). Post-hoc *t*-tests showed that overall sAA levels were significantly higher after the SECPT/WWT than before (s_2_–s_3_: *t*_(80)_ = −5.70, *p* < 0.001, *d* = 0.34, CI (0.03, 0.66)).

For cortisol, a main effect of the factor time (*F*_(2.1, 167.0)_ = 3.21, *p* < 0.001, η_p_^2^ = 0.12) and an interaction time × task (*F*_(2.1, 79)_ = 10.72, *p* < 0.001, η_p_^2^ = 0.12) were found. Separate rmANOVAs indicated that cortisol levels significantly changed during the experiment in both the SECPT (*F*_(2.0, 138.6)_ = 32.2, *p* < 0.001, η_p_^2^ = 0.32) and in the WWT group (*F*_(2.3,25.4)_ = 7.34, *p* = 0.002, η_p_^2^ = 0.40; Figure 3c). Post-hoc analyses indicated that cortisol levels significantly increased after the SECPT (s_2_–s_7_: *t*_(68)_ = −8.15, *p* < 0.001, *d* = 1.25, CI (−0.89, −0.54)) and did not significantly change after the WWT (s_2_–s_7_: *t*_(11)_ = 0.82, *p* = 0.431, *d* = 0.24, CI (−0.10, 0.21)).

Twelve participants (14.8%) were categorized as non-responders, 25 (30.9%) as only sAA responders, 17 (21.0%) as only cortisol responders, and 27 (33.3%) as sAA-and-cortisol responders. This classification was not significantly associated with the task that was performed (i.e., the SECPT or the WWT; Table 2). Mean perceived stress ratings, sAA, and cortisol levels for all responder groups are summarized in Table 3. None of the baseline variables (i.e., demographics, subjective ratings, physiological variables, and cognitive performance) differed significantly between the responder groups (*p* ≥ 0.072).

### 3.3. Memory Performance

**Figure 4:**
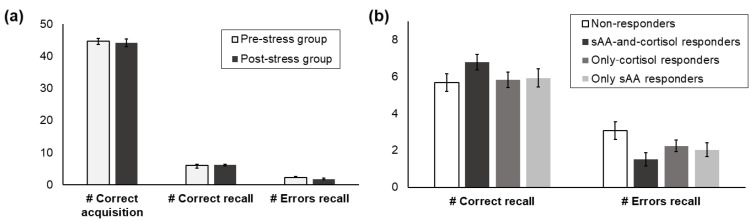
(**a**) Mean performance in the memory acquisition and the recall phase, separate for the pre- and the post-stress group (**a**) and the responder groups (**b**). The maximum number of correct items in the recall phase was nine. Standard errors are shown as error bars.

Memory recall performance and, therefore, implicit memory, did not significantly differ between the pre- and the post-stress groups (# Correct recall: *p* = 0.878, *d* = 0.03; # Errors recall: *p* = 0.225, *d* = 0.27; Figure 4a). Furthermore, implicit memory recall performance also did not differ between the responder groups (# Correct recall: *p* = 0.307; # Errors recall: *p* = 0.081; Figure 4b).

Additionally, we investigated whether performance during the acquisition phase differed between any of the groups. However, performance during the memory acquisition phase also did not significantly differ between the pre- and the post-stress groups (*p* = 0.763, *d* = 0.07) and also did not differ between the responder groups (*p* = 0.120).

### 3.4. Attentional Time Course

**Figure 5:**
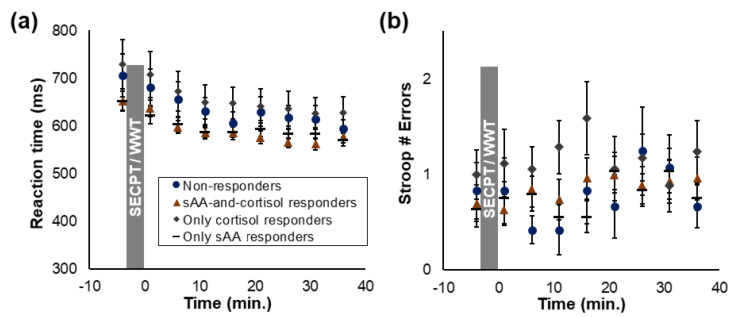
Attentional time course: (**a**) Reaction times and (**b**) number of errors in the Stroop task, separately for the responder groups. Standard errors are shown as error bars.

For the reaction times in the Stroop task, a main effect of the factor time (*F*_(4.37, 336.17)_ = 38.14, *p* < 0.001, η_p_^2^ = 0.33; Figure 5a), but no interaction time × responders (*p* = 0.677, η_p_^2^ = 0.03) was found, which reflected a decrease in reaction times throughout the experiment in all responder groups (Table 4). For the error rates in the Stroop task, neither a main effect of time (*p* = 0.466, η_p_^2^ = 0.01) nor an interaction time × responders (*p* = 0.644, η_p_^2^ = 0.03) was found, indicating that error rates did not change significantly throughout the experiment in all responder groups (Figure 5b). Thus, overall, no difference in the attentional time course between the responder groups was found.

### 3.5. Associations between Memory Recall and the Stress Response

#### 3.5.1. All Participants

Although no differences in memory performance could be found between the responder groups (Section 3.2), we performed additional analyses in which we investigated whether recall performance was related to the overall stress response, independent of the responder group. However, when using adjusted α-levels of α_adjusted_ = 0.002, no significant associations between any of the stress markers and recall performance were found. But, some associations were found (*p*_uncorrected_ < 0.05) that did, however, not pass the multiple comparison adjusted alpha level; the sAA-AUCg and the number of correctly recalled items (*r*_(__81)_ = −0.23, *p* = 0.036) as well as the percentage cortisol change and the number of errors during recall (*r*_(__81)_ = −0.24, *p* = 0.035) were negatively correlated.

#### 3.5.2. Pre- vs. Post-Stress Group

**Figure 6:**
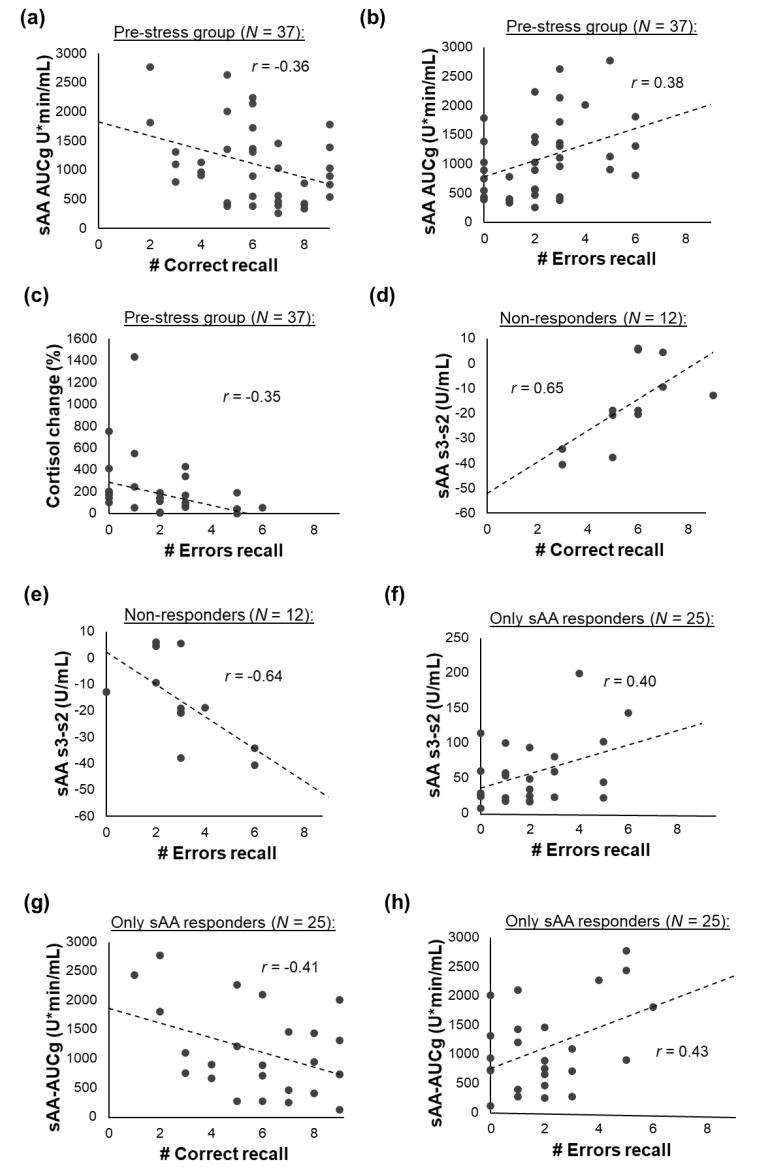
Associations between recall performance and markers for the stress response: (**a**–**c**) for the pre-stress group, (**d**,**e**) for the non-responders, and (**f**–**h**) for the only sAA responders. For the post-stress group, the only cortisol responders, and the sAA-and-cortisol responders, no associations were found.

Separate analyses of the pre- and the post-stress groups showed that these associations were only found for the pre-stress group (sAA-AUCg and # Correct recall: *r*_(__37)_ = −0.36, *p* = 0.027; cortisol percentage change and # Errors: *r*_(__37)_ = −0.35, *p* = 0.038: Figure 6a,c). Furthermore, a positive association between the sAA-AUCg and the number of errors in the recall phase was found for the pre-stress group (*r*_(__37)_ = 0.38, *p* = 0.021; Figure 6b). However, these correlations also did not survive the correction for multiple comparisons. In the post-stress group, no associations between the sAA- or cortisol response and memory performance were found (all *p* ≥ 0.338).

#### 3.5.3. Responder Groups

Sub-sample analyses for the responder groups also yielded no significant results when using adjusted α-levels. The following results were found for *p*_uncorrected_ = 0.05: For the non-responders, the sAA-concentration difference between s_2_ and s_3_ was positively associated with the number of correctly recalled items (*r*_(__12)_ = 0.65, *p* = 0.023) and negatively with the number of errors during recall (*r*_(__12)_ = −0.64, *p* = 0.027; Figure 6d,e). For the only-sAA-responders, the sAA-concentration difference between s_2_ and s_3_ was positively related with the number of errors during recall (*r*_(__25)_ = 0.40, *p* = 0.048; Figure 6f). Furthermore, the sAA-AUCg was positively related to the number of errors during recall (*r*_(__25)_ = 0.43, *p* = 0.032) and negatively related to the number of correctly recalled items (*r*_(__25)_ = −0.41, *p* = 0.040; Figure 6e,h). No associations between memory performance and the stress response were found for the only cortisol and the sAA-and-cortisol responders (all *p* ≥ 0.094).

### 3.6. Associations between Attention and Implicit Learning

#### 3.6.1. All Participants

Finally, we investigated whether implicit memory was associated with attentional performance during the experiment. However, we found that implicit memory recall performance (i.e., the number of correctly recalled items and the number of errors during recall) was not significantly associated with attentional performance at any of the time points (all *p* ≥ 0.111). Furthermore, we found that performance during the acquisition phase (i.e., during learning) was not significantly associated with attentional performance, i.e., it was not correlated with reaction times or error rates in the Stroop task at any of the time points (all *p* ≥ 0.155).

#### 3.6.2. Pre- vs. Post-Stress Group

**Figure 7:**
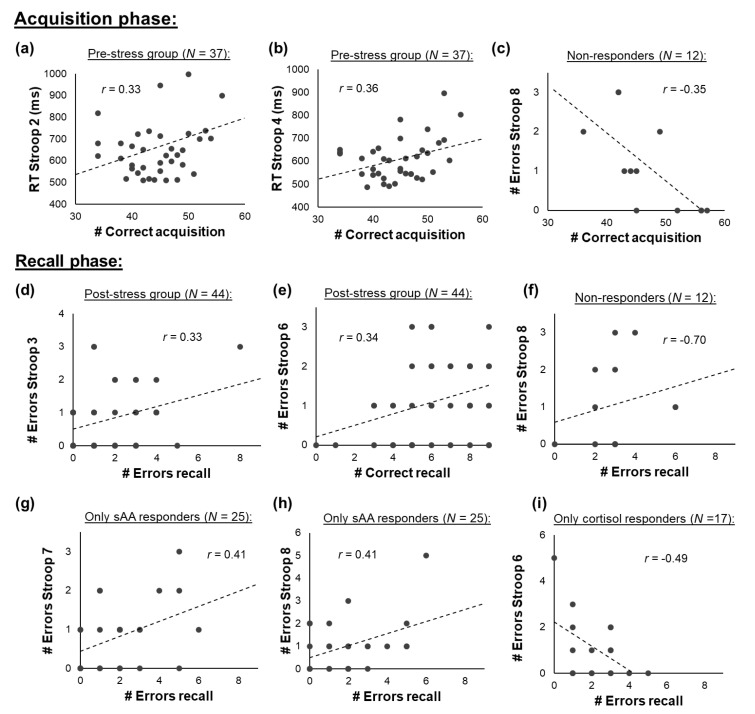
(**a**–**c**) Associations between attention and performance during the acquisition and (**d**–**i**) recall phase. No associations were found for the sAA-and-cortisol responders.

To test whether associations between the attention and implicit memory could be found for the pre- or the post stress group separately, additional analyses were performed. For both groups, no significant associations between Stroop performance and acquisition or recall performance were found when using the adjusted α-level. However, the following significant correlations were found for *p*_uncorrected_ = 0.05: for the pre-stress group, performance during the acquisition phase was positively correlated with the reaction time in the Stroop task immediately after the stressor/control (RT Stroop 2: *r*_(__37)_ = 0.33, *p* = 0.049) and ten minutes after the stressor (RT Stroop 4: *r*_(__37)_ = 0.36, *p* = 0.029; Figure 7a–c), indicating that a higher performance during the acquisition phase was associated with higher reaction times (lower attentional performance) after the stressor. No associations between recall performance and attention were found for the pre-stress group (all *p* ≥ 0.080). For the post-stress group, no associations between performance during the acquisition phase and attention were found (all *p* ≥ 0.238). However, the number of errors during recall was positively associated with the number of errors in the Stroop test five minutes after the stressor (# Errors Stroop 3: *r*_(__44)_ = 0.33, *p* = 0.030; Figure 7d), indicating that a lower recall performance was associated with a lower attentional performance five minutes after the stressor. Furthermore, attentional performance 20 min after the stressor was related to recall performance: more errors in the Stroop test were positively correlated with the number of correctly recalled items (# Errors Stroop 6: *r*_(__44)_ = 0.34, *p* = 0.026; Figure 7e) and negatively related with the number of errors during the recall phase (# Errors Stroop 6: *r*_(__44)_ = −0.34, *p* = 0.025).

#### 3.6.3. Responder Groups

Sub-sample analyses for the responder groups also yielded no significant associations between Stroop performance and acquisition or recall performance when using the adjusted α-level. The following significant correlations were found for *p*_uncorrected_ = 0.05: for the non-responders, the number of correctly recalled items was negatively associated with the number of errors 25 min after the stressor (#Errors Stroop 8: *r*_(__12)_ = −0.70, *p* = 0.011; Figure 7f), indicating that a better recall performance was associated with lower attentional performance afterwards. For the only sAA responders, positive associations between the number of errors during the recall phase and the number of errors in the Stroop task 25 min (# Errors Stroop 7: *r*_(25__)_ = 0.41, *p* = 0.042; Figure 7g) and 30 min after the stressor (# Errors Stroop 8: *r*_(__25)_ = 0.41, *p* = 0.041; Figure 7h) were found, indicating that participants with lower recall performance showed lower attentional performance afterwards. For the only cortisol responders, the number of errors during the recall phase was negatively correlated with the number of errors during the Stroop task 20 min after the stressor/control (# Errors Stroop 6: *r*_(__17)_ = −0.49, *p* = 0.045; Figure 7i), indicating that a better attentional performance was related to a lower recall performance at the same time point. For the sAA-and-cortisol responders, no associations between recall performance and attention were found (*p* ≥ 0.215).

## 4. Discussion

### 4.1. Summary and Discussion of Main Findings

The aim of the present study was to investigate the associations between the physiological acute stress response (i.e., catecholamine and glucocorticoid transmission) and implicit associative learning. Furthermore, the time course of attentional performance and its associations with the stress response and implicit learning were investigated. The results were complex. The main finding was that implicit memory recall did not significantly differ between those participants who were in the memory acquisition condition before they faced a stressor and those who were in the memory acquisition condition after they faced a stressor. There was also no significant difference in memory recall between the response groups (non-responders, only sAA responders, only cortisol responders, or sAA-and-cortisol responders). Therefore, our hypothesis that implicit memory recall performance would be associated with the acquisition time point (before or after the stressor/control) and would be better in the post-stress group than in the pre-stress group could not be confirmed. However, in previous studies in which an improvement has been reported, explicit memory tasks were used (e.g., [21,22]). Therefore, our findings might be specific for implicit memory tasks. One possible explanation for this lack of finding is that the time difference between the acquisition phases of both groups might not have been long enough. The acquisition phase of the post-stress group was performed right after the stressor, when sAA, but not cortisol levels, were high. Thus, SNS activation during memory acquisition might not be the crucial factor for memory improvement or impairment after acute stressors, which has been reported previously. Glucocorticoid transmission during the acquisition phase might be a more relevant factor. However, we are not able to answer this question with our study because cortisol levels did not significantly differ between the two acquisition time points. This explanation would be in line with the assumption that the hippocampus, which is sensitive to glucocorticoid responses, is a key structure for associative learning [3]. Therefore, other brain structures which are related with catecholamine responses (e.g., the prefrontal cortex or the locus coeruleus; [64,65,66]) do not seem to be of particular importance during the acquisition phase of the associative learning task that has been used in our study. However, this would be in contrast to the findings of Roebuck and colleagues, who found that acute stress, but not corticosterone injections, led to an improvement in associative learning in rats [67]. Therefore, the associations between glucocorticoid levels during the acquisition phase of implicit memory tasks should be further investigated in future research.

It should be noted that our memory task included an implicit acquisition phase, but that the learned associations were explicitly recalled. For a task that requires implicit recall (e.g., a perceptual priming task; [68]), a different pattern (i.e., an association between the stress response and memory recall) might be found.

### 4.2. Summary and Discussion of Further Findings

Besides our main findings, we performed some additional sub-sample analyses with regard to the associations between the acute stress response, implicit memory performance, and attention. These additional analyses yielded some results, however, they did not pass the multiple comparison adjusted alpha level, but showed large effect sizes (all *r* > 0.3): in the only sAA responders, who showed no cortisol increase, a steeper sAA response was associated with a lower recall performance. Furthermore, a lower recall performance (i.e., higher error rates during recall) was associated with lower attention at this time point (i.e., higher error rates in the Stroop task) in the only-sAA-responders. In contrast, the only cortisol responders, who showed no sAA response, showed the opposite pattern: a lower recall performance (i.e., more errors during recall) was associated with better attentional performance (i.e., lower error rates in the Stroop task) at this time point. No effects were found for the sAA-and-cortisol responders, which suggests that both effects are related to each other and might have canceled out each other.

Another interesting finding was that recall performance was associated with attention in the post-stress group only. Participants with higher attentional performance five minutes after the stressor (immediately after memory acquisition) showed better recall performance. In contrast, higher attentional performance at recall (20 min after acquisition) was associated with lower recall performance in the post-stress group.

For the non-responders, for whom no effects were hypothesized, some associations were found as well: Non-responders with higher sAA-decreases during the stressor showed lower recall performance. This suggests that a minimum level of catecholamines is beneficial for implicit learning. Furthermore, this finding is in line with previous studies that have shown the importance of physiological arousal and catecholamines for successful memory encoding and retrieval [69,70].

We conclude that the associations between sAA and cortisol responses with implicit learning are complex and are associated with each other. Further studies in which both (sAA and cortisol responses) are selectively (de-)activated are needed in order to get a deeper understanding. Overall, our study supports that implicit learning is related to attention, which has been suggested previously [37,38,39]. An additional aim of our study was to investigate the role of general cognitive functioning (i.e., baseline attentional performance) for implicit learning under stress. Since we found no associations between baseline Stroop performance and implicit memory, we conclude that this was not a considerable factor in our study.

### 4.3. Physiological Responses to the Stress and the Control Task

We used a stress and a control task (the SECPT and the WWT) because we intended to induce different response patterns (the four responder groups), which should include a non-responder group. Overall, the typical sAA- and cortisol-response patterns were found for the SECPT: sAA levels peaked immediately after the stressor and cortisol levels were highest, 20 min after it. For the WWT, an unexpectedly high number of responders was found as well. From both groups together, participants were categorized as non-responders, only sAA responders, only cortisol responders, and sAA-and-cortisol responders. However, because of the overall high respondence rates, 12 participants were classified as non-responders only, although 12 participants took part in the control condition. Most of the participants from the control condition were categorized as only sAA responders and only one participant from the WWT group showed a cortisol response. Therefore, the reason for the stress response in the control condition was probably not due to social stress which typically leads to strong cortisol responses [71,72,73]. It is more likely that either the water was too warm and might have introduced a SNS response or—more likely—the cognitive testing throughout the session might have stressed the participants [74,75]. However, throughout the experiment, participants became faster during the Stroop task, which does not necessarily indicate stress effects, but is probably due to learning effects.

### 4.4. Limitations and Directions for Future Research

Besides the low number of non-responders and the presumably continuous additional stress from the cognitive testing, our study is subject to some further limitations. The most important of these is the timeline of the acquisition phase, which should be moved to later time points (e.g., to the cortisol peak) in future studies. This would allow an investigation of the associations between the acquisition of implicit memory and the glucocorticoid response. Furthermore, all the associations reported and discussed in Section 4.2 should be interpreted with caution because they did not pass the correction for multiple comparisons, although they showed large effect sizes. Another limitation is that the time delay between memory acquisition and recall was about 25 min in the pre-stress and about 20 min in the post-stress group, which might be another explanation as to why we did not find a difference in memory recall between both groups. Moreover, the memory task that was used in our study is not generalizable to other implicit memory tasks (e.g., serial implicit learning, forced choice recognition, or priming tasks), which should also be investigated in future research. Another limitation is that our memory task included an implicit acquisition phase, but the learned associations were explicitly recalled. Therefore, future studies are needed in which both acquisition and recall are implicitly tested. To achieve this goal, a perceptual priming task [68,76] could be used.

In our study, the Stroop task was used for assessing attention and the baseline levels were used as measures for general attentional ability. The changes in this (general) attentional performance were used as indirect measures for attentional processes. However, there are other methods (e.g., electroencephalography or pupillometry, e.g., [77,78]) with which attentional processing would have been assessable more directly and for which additional tasks, which might have stressed the participants, would not have been necessarily needed.

Furthermore, our findings are not generalizable to other target groups (e.g., children, older people, or clinical samples) and other settings. Future research should focus on other groups and on the effects of acute stress on implicit learning in field studies (e.g., in schools or other learning environments).

## 5. Conclusions

To summarize, the main finding of our study was that associative memory recall was not significantly associated with the acquisition time point (i.e., pre or post stressor) and was, therefore, not significantly associated with the catecholamine response. Furthermore, no differences between the responder groups (i.e., non-responders, sAA-and-cortisol responders, only sAA responders, and only cortisol responders) were found, neither for recall performance nor for the attentional time course during the experiment. However, sub-group analyses indicated (although this did not pass the multiple comparison adjusted alpha level) that in the only sAA responders, a steeper sAA response was associated with a lower recall performance. Furthermore, a lower recall performance was associated with lower attention in this group. In contrast, the only cortisol responders showed the opposite pattern: a lower recall performance was associated with higher attentional performance. We conclude that the associations of sAA and cortisol responses with implicit associative learning and memory are complex and are related to each other. Further studies in which both (catecholamine and glucocorticoid responses) are selectively (de-)activated are needed. General (baseline) attentional performance was not a considerable factor for implicit memory in our study. Therefore, we conclude that attention, although related with implicit learning and memory, is not a protective factor for implicit associative learning under stress. In future studies, different implicit learning tasks and less, potentially stressful, attentional assessments should be used. Furthermore, field studies in which the associations between acute stress and implicit associative learning are investigated in everyday life are needed.

## Figures and Tables

**Table 1:** Participants’ demographic characteristics and baseline (BL) stress and cognitive levels (mean (M) and standard deviation (SD)) for the whole sample and separately for the warm-water test (WWT) and the socially evaluated cold-pressor test (SECPT) group as well as for the pre-stress and the post-stress group.

	Overall (*N* = 81)		WWT (*N* = 12)		SECPT (*N* = 69)		Pre-Stress (*N* = 37)		Post-Stress (*N* = 44)	
	*N*	%	*N*	%	*N*	%	*N*	%	*N*	%
Female	56	69.1	6	50	50	72.5	27	73	29	65.9
Smokers	2	2.5	1	8.3	1	1.4	2	5.4	0	0
	**M**	**SD**	**M**	**SD**	**M**	**SD**	**M**	**SD**	**M**	**SD**
Age (years)	21.8	3.9	22.8	3.6	21.6	4.0	21.9	4.0	21.6	4.0
BMI ^1^ (kg/m^2^)	22.1	2.8	23.3	3.4	21.8	2.6	22.3	2.9	21.8	2.7
BL stress rating	2.8	1.3	2.8	1.7	2.8	1.3	2.6	1.5	2.9	1.2
BL sAA ^2^ (U/mL)	109.7	71.0	100.4	49.8	111.4	74.2	108.6	68.0	110.7	74.1
BL cortisol (nmol/L)	2.9	2.0	3.6	1.9	2.7	2.0	3.0	2.3	2.8	1.7
BL Stroop reaction time (ms)	677.3	142.7	711.1	152.9	671.4	141.2	676.2	149.8	678.2	138.2
BL Stroop errors	0.8	1.0	0.8	1.1	0.8	1.0	0.8	1.1	0.7	0.9

^1^ BMI: body mass index, ^2^ sAA: salivary alpha-amylase.

**Table 2:** Number of participants who were categorized as non-responders, only sAA responders, only cortisol responders, and sAA-and-cortisol responders with respect to the performed stress/control task.

	SECPT ^1^	WWT ^2^	*N*
Non-responders	9	3	12
Only sAA responders	17	8	25
Only cortisol responders	16	1	17
sAA-and-cortisol responders	27	0	27
Overall	69	12	81

^1^ SECPT: socially evaluated cold-pressor test, ^2^ WWT: warm-water test.

**Table 3:** Mean perceived stress ratings, sAA and cortisol levels for the response categories for the eleven time points.

	Overall		Non-Responders		sAA-and-Cortisol Responders		Only Cortisol Responders		Only sAA Responders	
	(*N* = 81)		(*N* = 12)		(*N* = 27)		(*N* = 17)		(*N* = 25)	
	Mean	SD	Mean	SD	Mean	SD	Mean	SD	Mean	SD
Rating s1	2.8	1.3	2.7	1.5	2.7	1.4	2.9	1.3	2.8	1.3
Rating s2	3.4	1.7	2.8	1.4	3.0	1.6	3.7	1.7	4.0	1.9
Rating s3	3.8	2.0	3.3	1.9	3.5	1.7	4.8	2.2	3.6	2.2
Rating s4	3.1	1.5	2.8	1.1	3.0	1.4	3.8	2.0	2.9	1.3
Rating s5	2.6	1.5	2.3	1.1	2.4	1.4	3.3	2.0	2.4	1.3
Rating s6	2.4	1.4	2.2	0.8	2.1	1.1	2.9	1.7	2.3	1.5
Rating s7	2.2	1.2	1.9	1.0	2.0	1.1	2.5	1.4	2.2	1.1
Rating s8	2.0	1.1	1.9	1.2	1.9	0.9	2.2	1.2	2.1	1.2
Rating s9	1.9	0.9	2.0	1.0	1.7	0.7	2.2	1.1	1.8	1.0
Rating s10	1.9	1.0	2.1	1.4	1.7	0.8	1.9	1.1	1.8	1.0
Rating s11	1.7	0.8	1.8	1.0	1.7	0.8	1.6	0.7	1.7	0.9
sAA s1	109.7	71.0	81.4	47.5	108.3	57.4	136.8	96.2	106.5	71.3
sAA s2	94.2	71.2	107.0	68.5	79.2	46.5	137.7	101.9	74.7	58.5
sAA s3	124.0	79.6	90.7	63.5	138.7	64.7	112.9	94.5	131.6	88.6
sAA s4	119.7	81.6	82.0	36.7	125.2	70.7	149.0	115.9	112.0	76.0
sAA s5	106.2	71.7	68.0	31.4	109.6	64.9	138.8	93.9	98.6	68.3
sAA s6	89.7	61.4	56.6	34.9	96.6	56.6	111.1	76.2	83.5	60.7
sAA s7	102.6	67.4	79.2	46.0	105.4	52.9	136.6	101.7	87.6	53.8
sAA s8	104.3	69.0	81.2	41.6	106.0	55.7	132.9	100.2	94.1	63.6
sAA s9	106.2	74.6	83.2	52.8	105.2	53.8	145.2	116.6	91.8	59.4
sAA s10	93.2	70.0	91.3	57.7	88.4	54.2	129.1	104.8	74.7	54.8
sAA s11	107.7	72.9	91.9	76.0	107.0	55.6	145.8	103.6	90.0	55.7
Cortisol s1	2.9	2.0	3.3	2.5	2.4	1.4	3.8	2.3	2.5	1.7
Cortisol s2	2.7	1.9	2.9	1.8	2.1	1.2	4.0	2.8	2.2	1.4
Cortisol s3	3.0	2.6	3.1	2.4	2.4	1.4	5.0	4.1	2.3	1.5
Cortisol s4	3.2	3.3	2.6	1.6	2.6	1.6	5.8	6.0	2.2	1.3
Cortisol s5	4.2	4.4	2.5	1.6	4.5	2.9	7.8	7.6	2.3	1.4
Cortisol s6	5.1	5.3	2.3	1.1	6.7	4.1	8.8	8.4	2.2	1.4
Cortisol s7	6.0	6.7	2.5	1.4	7.9	4.9	11.2	10.8	2.1	1.3
Cortisol s8	5.3	5.5	2.4	1.2	6.9	4.9	9.8	7.8	1.9	1.1
Cortisol s9	5.2	5.5	2.9	2.4	6.7	4.9	9.3	8.2	1.9	1.1
Cortisol s10	4.6	4.8	2.4	1.7	5.6	3.7	8.6	7.4	1.7	0.9
Cortisol s11	4.1	3.9	2.2	1.3	4.8	3.2	7.5	5.5	1.7	0.8

**Table 4:** Mean reaction times (RT) and number of errors in the Stroop task.

	Overall		Non-Responders		sAA-and-Cortisol Responders		Only Cortisol Responders		Only sAA Responders	
	(*N* = 81)		(*N* = 12)		(*N* = 27)		(*N* = 17)		(*N* = 25)	
	Mean	SD	Mean	SD	Mean	SD	Mean	SD	Mean	SD
RT 1 (ms)	677.3	142.7	706.8	154.3	652.7	108.6	729.9	212.9	653.9	101.5
RT 2 (ms)	654.7	128.0	682.3	134.4	637.6	87.9	708.7	198.9	623.2	88.1
RT 3 (ms)	624.8	112.9	656.5	130.9	598.3	71.7	673.6	169.9	604.8	80.4
RT 4 (ms)	606.7	93.2	631.9	97.0	585.4	62.0	650.7	147.9	587.8	57.8
RT 5 (ms)	602.8	89.2	606.8	80.6	585.4	68.5	648.2	138.8	588.7	60.0
RT 6 (ms)	604.3	100.9	630.8	108.8	576.7	70.0	642.6	147.6	595.2	79.8
RT 7 (ms)	594.2	91.8	618.9	87.5	565.4	53.5	637.2	152.9	584.4	56.9
RT 8 (ms)	590.7	88.6	614.9	100.5	561.9	61.1	626.6	135.4	585.9	55.8
RT 9 (ms)	588.9	84.8	594.6	64.6	577.5	60.9	629.0	135.2	571.4	65.2
Errors 1	0.8	1.0	0.8	1.0	0.7	1.0	1.0	1.1	0.6	1.0
Errors 2	0.8	1.0	0.8	1.2	0.6	0.8	1.1	1.5	0.8	0.8
Errors 3	0.8	0.9	0.4	0.5	0.9	1.0	1.1	1.0	0.8	0.9
Errors 4	0.8	1.0	0.4	0.9	0.7	1.1	1.3	1.2	0.6	0.7
Errors 5	1.0	1.2	0.8	1.2	1.0	1.1	1.6	1.6	0.6	0.8
Errors 6	1.0	1.1	0.7	1.2	1.0	1.0	1.1	1.4	1.0	1.0
Errors 7	1.0	1.1	1.3	1.6	0.9	1.0	1.2	1.0	0.8	0.9
Errors 8	1.0	1.1	1.1	1.2	0.9	1.2	0.9	1.1	1.0	1.2
Errors 9	0.9	1.0	0.7	0.8	1.0	1.2	1.2	1.3	0.8	0.7

## References

[1] Squire L.R., Zola-Morgan S. (1988). Memory: Brain systems and behavior. Trends Neurosci..

[2] Henke K. (2010). A model for memory systems based on processing modes rather than consciousness. Nat. Rev. Neurosci..

[3] Henke K., Buck A., Weber B., Wieser H.G. (1997). Human hippocampus establishes associations in memory. Hippocampus.

[4] Preston A.R., Gabrieli J.D.E. (2008). Dissociation between explicit memory and configural memory in the human medial temporal lobe. Cereb. Cortex.

[5] Schott B.H., Sellner D.B., Lauer C.-J., Habib R., Frey J.U., Guderian S., Heinze H.-J., Düzel E. (2004). Activation of midbrain structures by associative novelty and the formation of explicit memory in humans. Learn. Mem..

[6] Luethi M., Meier B., Sandi C. (2008). Stress effects on working memory, explicit memory, and implicit memory for neutral and emotional stimuli in healthy men. Front. Behav. Neurosci..

[7] Ehlers M., Todd R. (2017). Acute psychophysiological stress impairs human associative learning. Neurobiol. Learn. Mem..

[8] Meyer T., Smeets T., Giesbrecht T., Quaedflieg C.W.E.M., Merckelbach H. (2013). Acute stress differentially affects spatial configuration learning in high and low cortisol-responding healthy adults. Eur. J. Psychotraumatology.

[9] Chrousos G.P. (1992). The concepts of stress and stress system disorders. Overview of physical and behavioral homeostasis. JAMA.

[10] Stratakis C.A., Chrousos G.P. (1995). Neuroendocrinology and pathophysiology of the stress system. Ann. N. Y. Acad. Sci..

[11] Arnsten A.F.T. (2009). Stress signalling pathways that impair prefrontal cortex structure and function. Nat. Rev. Neurosci..

[12] Ramos B.P., Arnsten A.F.T. (2007). Adrenergic pharmacology and cognition: Focus on the prefrontal cortex. Pharmacol. Ther..

[13] Lupien S., Maheu F., Tu M., Fiocco A., Schramek T. (2007). The effects of stress and stress hormones on human cognition: Implications for the field of brain and cognition. Brain Cogn..

[14] De Kloet E.R., Reul J.M., Sutanto W. (1990). Corticosteroids and the brain. J. Steroid Biochem. Mol. Boil..

[15] Lupien S.J., De Leon M.J., De Santi S., Convit A., Tarshish C., Nair N.P.V., Thakur M., McEwen B.S., Hauger R.L., Meaney M.J. (1998). Cortisol levels during human aging predict hippocampal atrophy and memory deficits. Nat. Neurosci..

[16] Buchanan T.W., Lovallo W.R. (2001). Enhanced memory for emotional material following stress-level cortisol treatment in humans. Psychoneuroendocrinology.

[17] Joëls M., Fernández G., Roozendaal B. (2011). Stress and emotional memory: A matter of timing. Trends Cogn. Sci..

[18] Wolf O. (2017). Stress and memory retrieval: Mechanisms and consequences. Curr. Opin. Behav. Sci..

[19] Kirschbaum C., Wolf O., May M., Wippich W., Hellhammer D. (1996). Stress and treatment-induced elevations of cortisol levels associated with impaired declarative memory in healthy adults. Life Sci..

[20] Roozendaal B., Okuda S., De Quervain D.-F., McGaugh J. (2006). Glucocorticoids interact with emotion-induced noradrenergic activation in influencing different memory functions. Neuroscience.

[21] Barsegyan A., MacKenzie S.M., Kurose B.D., McGaugh J.L., Roozendaal B. (2010). Glucocorticoids in the prefrontal cortex enhance memory consolidation and impair working memory by a common neural mechanism. Proc. Natl. Acad. Sci. USA.

[22] Smeets T., Otgaar H., Candel I., Wolf O. (2008). True or false? Memory is differentially affected by stress-induced cortisol elevations and sympathetic activity at consolidation and retrieval. Psychoneuroendocrinology.

[23] Beckner V., Tucker D.M., Delville Y., Mohr D. (2006). Stress facilitates consolidation of verbal memory for a film but does not affect retrieval. Behav. Neurosci..

[24] Schwabe L., Wolf O. (2013). Stress and multiple memory systems: From ‘thinking’ to ‘doing’. Trends Cogn. Sci..

[25] Wong T.P., Howland J.G., Robillard J.M., Ge Y., Yu W., Titterness A.K., Brebner K., Liu L., Weinberg J., Christie B.R. (2007). Hippocampal long-term depression mediates acute stress-induced spatial memory retrieval impairment. Proc. Natl. Acad. Sci. USA.

[26] Oei N.Y.L., Everaerd W.T.A.M., Elzinga B.M., Van Well S., Bermond B. (2006). Psychosocial stress impairs working memory at high loads: An association with cortisol levels and memory retrieval. Stress.

[27] Gagnon S.A., Wagner A.D. (2016). Acute stress and episodic memory retrieval: Neurobiological mechanisms and behavioral consequences. Ann. N. Y. Acad. Sci..

[28] Smeets T. (2011). Acute stress impairs memory retrieval independent of time of day. Psychoneuroendocrinology.

[29] Dinse H.R., Kattenstroth J., Lenz M., Tegenthoff M., Wolf O. (2017). The stress hormone cortisol blocks perceptual learning in humans. Psychoneuroendocrinology.

[30] Dierolf A.M., Fechtner J., Böhnke R., Wolf O., Naumann E. (2017). Influence of acute stress on response inhibition in healthy men: An ERP study. Psychophysiology.

[31] Schoofs D., Preuß D., Wolf O. (2008). Psychosocial stress induces working memory impairments in an n-back paradigm. Psychoneuroendocrinology.

[32] Becker L., Rohleder N. (2019). Time course of the physiological stress response to an acute stressor and its associations with the primacy and recency effect of the serial position curve. PLoS ONE.

[33] Vedhara K., Hyde J., Gilchrist I.D., Tytherleigh M., Plummer S. (2000). Acute stress, memory, attention and cortisol. Psychoneuroendocrinology.

[34] Shields G.S., Bonner J.C., Moons W.G. (2015). Does cortisol influence core executive functions? A meta-analysis of acute cortisol administration effects on working memory, inhibition, and set-shifting. Psychoneuroendocrinology.

[35] Chun M.M., Turk-Browne N.B. (2007). Interactions between attention and memory. Curr. Opin. Neurobiol..

[36] Seger C.A. (1994). Implicit learning. Psychol. Bull..

[37] Rausei V., Makovski T., Jiang Y.V. (2007). Attention dependency in implicit learning of repeated search context. Q. J. Exp. Psychol..

[38] Chun M.M., Jiang Y. (1998). Contextual cueing: Implicit learning and memory of visual context guides spatial attention. Cogn. Psychol..

[39] Jiang Y., Chun M.M. (2001). Selective attention modulates implicit learning. Q. J. Exp. Psychol. Sect. A.

[40] Chajut E., Algom D. (2003). Selective attention improves under stress: Implications for theories of social cognition. J. Pers. Soc. Psychol..

[41] Booth R., Sharma D. (2009). Stress reduces attention to irrelevant information: Evidence from the Stroop task. Motiv. Emot..

[42] Sänger J., Bechtold L., Schoofs D., Blaszkewicz M., Wascher E. (2014). The influence of acute stress on attention mechanisms and its electrophysiological correlates. Front. Behav. Neurosci..

[43] Belanoff J.K., Gross K., Yager A., Schatzberg A.F. (2001). Corticosteroids and cognition. J. Psychiatr. Res..

[44] Lupien S.J., Gillin C.J., Hauger R.L. (1999). Working memory is more sensitive than declarative memory to the acute effects of corticosteroids: A dose-response study in humans. Behav. Neurosci..

[45] Putman P., Hermans E.J., Van Honk J. (2010). Cortisol administration acutely reduces threat-selective spatial attention in healthy young men. Physiol. Behav..

[46] Stroop J.R. (1935). Studies of interference in serial verbal reactions. J. Exp. Psychol..

[47] Wechsler D. (1955). Manual for the Wechsler Adult Intelligence Scale.

[48] Becker L., Schade U., Rohleder N. (2019). Evaluation of the socially evaluated cold-pressor group test (SECPT-G) in the general population. PeerJ.

[49] Minkley N., Schröder T.P., Wolf O., Kirchner W.H. (2014). The socially evaluated cold-pressor test (SECPT) for groups: Effects of repeated administration of a combined physiological and psychological stressor. Psychoneuroendocrinology.

[50] Schwabe L., Haddad L., Schachinger H. (2008). HPA axis activation by a socially evaluated cold-pressor test. Psychoneuroendocrinology.

[51] Nater U.M., Rohleder N., Schlotz W., Ehlert U., Kirschbaum C. (2007). Determinants of the diurnal course of salivary alpha-amylase. Psychoneuroendocrinology.

[52] Thoma M.V., Kirschbaum C., Wolf J.M., Rohleder N. (2012). Acute stress responses in salivary alpha-amylase predict increases of plasma norepinephrine. Boil. Psychol..

[53] Bosch J.A., De Geus E., Veerman E.C.I., Hoogstraten J., Amerongen A.V.N. (2003). Innate secretory immunity in response to laboratory stressors that evoke distinct patterns of cardiac autonomic activity. Psychosom. Med..

[54] Rohleder N., Nater U.M. (2009). Determinants of salivary α-amylase in humans and methodological considerations. Psychoneuroendocrinology.

[55] Miller R., Plessow F., Kirschbaum C., Stalder T. (2013). Classification Criteria for distinguishing cortisol responders from nonresponders to psychosocial stress. Psychosom. Med..

[56] Schwabe L., Schächinger H. (2018). Ten years of research with the Socially Evaluated Cold Pressor Test: Data from the past and guidelines for the future. Psychoneuroendocrinology.

[57] Pruessner J.C., Kirschbaum C., Meinlschmid G., Hellhammer D.H. (2003). Two formulas for computation of the area under the curve represent measures of total hormone concentration versus time-dependent change. Psychoneuroendocrinology.

[58] Mauchly J.W. (1940). Significance test for sphericity of a normal *n*-variate distribution. Ann. Math. Stat..

[59] Greenhouse S.W., Geisser S. (1959). On methods in the analysis of profile data. Psychometrika.

[60] Morris S.B. (2007). Estimating effect sizes from pretest-posttest-control group designs. Organ. Res. Methods.

[61] Sinclair J., Taylor P.J., Hobbs S.J. (2013). Alpha level adjustments for multiple dependent variable analyses and their applicability—A review. Int. J. Sports Sci. Eng..

[62] Hubbard R., Lindsay R.M. (2008). Why *p* values are not a useful measure of evidence in statistical significance testing. Theory Psychol..

[63] Gignac G., Szodorai E.T. (2016). Effect size guidelines for individual differences researchers. Pers. Individ. Differ..

[64] Sara S.J. (2009). The locus coeruleus and noradrenergic modulation of cognition. Nat. Rev. Neurosci..

[65] Foote S.L., Morrison J.H. (1987). Extrathalamic modulation of cortical function. Annu. Rev. Neurosci..

[66] Valentino R.J., Van Bockstaele E. (2008). Convergent regulation of locus coeruleus activity as an adaptive response to stress. Eur. J. Pharmacol..

[67] Roebuck A.J., Liu M.C., Lins B.R., Scott G.A., Howland J.G. (2018). Acute stress, but not corticosterone, facilitates acquisition of paired associates learning in rats using touchscreen-equipped operant conditioning chambers. Behav. Brain Res..

[68] Schacter D.L. (1992). Priming and multiple memory systems: Perceptual mechanisms of implicit memory. J. Cogn. Neurosci..

[69] Cahill L., Prins B., Weber M., McGaugh J.L. (1994). β-Adrenergic activation and memory for emotional events. Nature.

[70] Schoenfeld B.J. (2013). Potential mechanisms for a role of metabolic stress in hypertrophic adaptations to resistance training. Sports Med..

[71] Dickerson S.S., Gruenewald T.L., Kemeny M.E. (2004). When the social self is threatened: Shame, physiology, and health. J. Pers..

[72] Dickerson S.S., Kemeny M.E. (2004). Acute stressors and cortisol responses: A theoretical integration and synthesis of laboratory research. Psychol. Bull..

[73] Gruenewald T.L., Dickerson S.S., Kemeny M.E., Tracy J.L., Robins R.W., Tangney J.P. (2007). The Self-Conscious Emotions: Theory and Research.

[74] Skoluda N., Strahler J., Schlotz W., Niederberger L., Marques S., Fischer S., Thoma M.V., Spoerri C., Ehlert U., Nater U.M. (2015). Intra-individual psychological and physiological responses to acute laboratory stressors of different intensity. Psychoneuroendocrinology.

[75] Becker L., Schade U., Rohleder N. (2020). Activation of the hypothalamic-pituitary adrenal axis in response to a verbal fluency task and associations with task performance. PLoS ONE.

[76] Musen G., Treisman A. (1990). Implicit and explicit memory for visual patterns. J. Exp. Psychol. Learn. Mem. Cogn..

[77] Unsworth N., Robison M.K. (2017). The importance of arousal for variation in working memory capacity and attention control: A latent variable pupillometry study. J. Exp. Psychol. Learn. Mem. Cogn..

[78] Klimesch W., Doppelmayr M., Russegger H., Pachinger T., Schwaiger J. (1998). Induced alpha band power changes in the human EEG and attention. Neurosci. Lett..

